# MXene based nanocomposite films

**DOI:** 10.1002/EXP.20220049

**Published:** 2022-07-18

**Authors:** Lei Li, Qunfeng Cheng

**Affiliations:** ^1^ School of Chemistry Key Laboratory of Bio‐Inspired Smart Interfacial Science and Technology of Ministry of Education Beihang University Beijing China; ^2^ School of Materials Science and Engineering Zhengzhou University Zhengzhou China

**Keywords:** electromagnetic interference shielding, mechanical property, MXene, nanocomposite films, sequential bridging

## Abstract

Since the first report in 2011, two‐dimensional transition metal carbide/nitride MXenes have aroused widespread attention owing to the particular structure and physiochemical properties. In the last few years, MXene‐based nanocomposite films have been widely investigated, showing promising applications in many fields. However, poor mechanical properties and thermal/electrical conductivities of MXene‐based nanocomposite films still limited their practical applications. Herein, we summarize the fabrication approach of MXene‐based nanocomposite films and discuss the mechanical properties and other applications, including electromagnetic interference shielding, thermal conductivity, and supercapacitors. Then, several vital factors for fabricating high performance MXene based nanocomposite films have been refined. To further fabricate high performance MXene‐based nanocomposite films, some effective sequential bridging strategies are also discussed. Lastly, a conclusion of the challenges and opportunities of MXene‐based nanocomposite films is provided to facilitate their development and application for various purposes in the future of scientific research.

## Graphical abstract

This review summarized the fabrication method of MXene‐based nanocomposite films and discussed the mechanical properties and the application of electromagnetic shielding, thermal conductivity, and supercapacitors. Finally, based on the summary of previous works, we prospected the challenges and prospects of MXene‐based nanocomposite films in the future of scientific research.

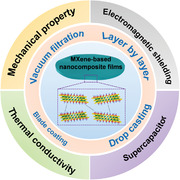

## 1 INTRODUCTION

MXenes are novel transition metal carbides or nitrides synthesized through selectively etching A layers of the layered carbides or nitrides which are named MAX phases.^[^ ^1^, ^2^, ^3^, ^4^ ^]^ The typically chemical formula is M*_n_*_+ 1_AX*_n_*, in which the *M* is a transition metal, *A* is an element from groups III;A and IVA, X is nitrogen or carbon and *n* = 1, 2, 3, and 4.^[^ ^5^ ^]^ In addition, the obtained MXene has the chemical formula M_*n* + 1_X*_n_* T*_x_*, where T*_x_* corresponds to the surface functional terminating groups such as ‐F, ‐O, and ‐OH.^[^ ^1^ ^]^ Because of the promising mechanical properties,^[^ ^6^ ^]^ electrical^[^ ^7^ ^]^ and thermal conductivities,^[^ ^8^ ^]^ MXene‐based nanocomposite films have been widely investigated and used in flexible and electronical devices and aerospace, such as electromagnetic interference (EMI) shielding,^[^ ^9^ ^]^ thermal conductivity,^[^ ^10^ ^]^ and supercapacitors,^[^ ^11^ ^]^ respectively. Furthermore, assembling MXene flakes into high performance macroscopic films is essential to realize these applications.

Since 2011, there have been many studies on MXene‐based nanocomposite films.^[^ ^12^, ^13^, ^14^, ^15^ ^]^ However, during the assembly process of MXene‐based nanocomposite films, the voids have been formed inevitably, which leads to the poor performance compared to the intrinsic properties of MXene. Hence, how to eliminate the existing voids is a crucial factor for fabricating high performance MXene‐based nanocomposite films. The weak interface interactions between MXene flakes are still needed to be strengthened by other fillers via hydrogen bonding, covalent bonding, or ionic bonding.^[^ ^16^, ^17^ ^]^ Furthermore, aligning MXene flakes plays another role in enhancing the performance of MXene‐based nanocomposite films. Thus, the compactness, interlayer interaction, and alignment are three vital basics for realizing the high performance of MXene‐based nanocomposite films.

Herein, we summarize latest researches on MXene‐based nanocomposite films. We start with a concise review of the fabrication process of MXene‐based nanocomposite films, then we classify the kinds of methods. We also discuss the mechanical properties, and highlight the applications of EMI shielding, thermal conductivity, and supercapacitor. In conclusion, we generalize current challenges and propose prospect research directions for vigorous progress of nanocomposite films based on MXene.

## 2 FABRICATION PROCESS OF MXene BASED NANOCOMPOSITE FILMS

Due to the unique 2D lamellar structure, active terminal groups and excellent mechanical and electrical properties, MXene is considered as a prospective candidate for constructing multifunctional nanocomposite films. Up to now, there are many studies about MXene‐based nanocomposite films via some fabrication approaches such as vacuum‐assisted filtration (VAF),^[^ ^9^, ^12^, ^17^, ^18^, ^19^, ^20^, ^21^, ^22^ ^]^ layer‐by‐layer (LbL),^[^ ^23^, ^24^, ^25^ ^]^ blade coating,^[^ ^26^ ^]^ and drop casting,^[^ ^27^ ^]^ and so forth.

**FIGURE 1:**
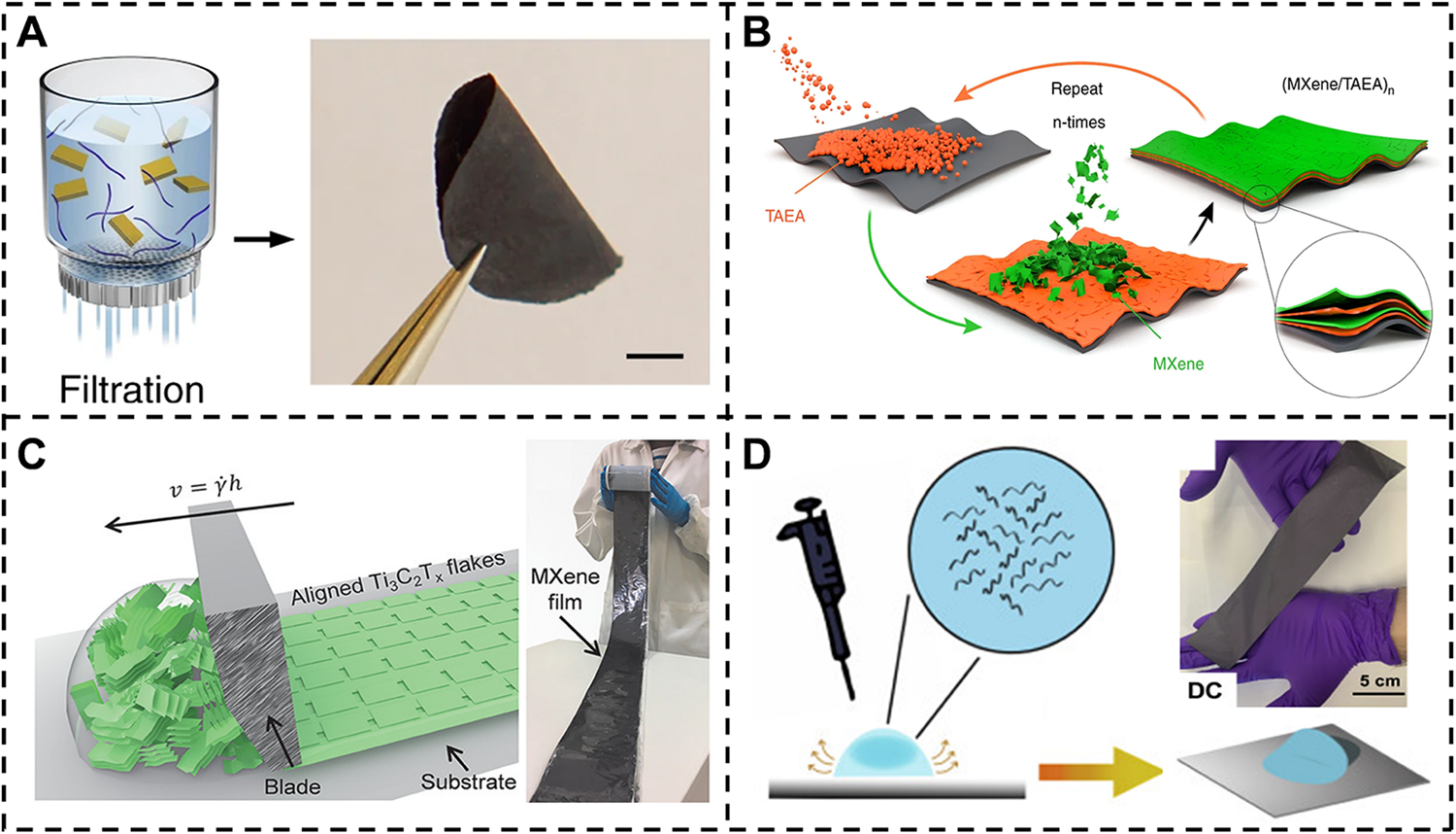
Fabricating process of MXene‐based nanocomposite films. (A) Schematic diagram of the vacuum‐assisted filtration approach for fabricating MXene‐ANF nanocomposite films. Reproduced with permission.^[^ ^19^ ^]^ Copyright 2019, Springer Nature. (B) Schematic diagram of the (MXene/TAEA)*_n_* multilayers nanocomposites by LbL assembly. Reproduced with permission.^[^ ^23^ ^]^ Copyright 2019, Springer Nature. (C) Schematic diagram of the blade coating approach for fabricating MXene films. Reproduced with permission.^[^ ^26^ ^]^ Copyright 2020, Wiley‐VCH. (D) Schematic diagram of the fabrication approach for the drop‐casting MXene films. Reproduced with permission.^[^ ^27^ ^]^ Copyright 2020, Elsevier

VAF is a widely used technique for fabricating free‐standing films from assembling 2D nanosheets (Figure 1A).^[^ ^19^ ^]^ Since 2014, Ling et al.^[^ ^12^ ^]^ reported the flexible Ti_3_C_2_T*_x_* MXene film with outstanding electrochemical performance via VAF. After that, enormous MXene nanocomposite films have been constructed with adding various filler materials, such as polyvinyl alcohol (PVA),^[^ ^12^ ^]^ cellulose nanofiber (CNF),^[^ ^28^ ^]^ poly(diallyldimethylammonium chloride) (PDDA),^[^ ^12^ ^]^ aramid nanofiber (ANF),^[^ ^29^ ^]^ carbon nanotubes (CNTs),^[^ ^22^ ^]^ sodium alginate (SA),^[^ ^17^ ^]^ poly(3,4‐ethylenedioxythiophene)−poly‐(styrenesulfonate) (PEDOT:PSS),^[^ ^30^ ^]^ bacterial cellulose (BC),^[^ ^31^ ^]^ carboxymethyl cellulose (CMC),^[^ ^20^ ^]^ graphene oxide (GO),^[^ ^32^ ^]^ nano clay,^[^ ^33^ ^]^ and boron nitride (BN).^[^ ^34^ ^]^ Except the simple VAF technique, the LbL self‐assembly is a prevalent fabrication approach for assembling multilayer nanocomposite films of desired thickness especially for 2D nanomaterials. For instance, Tian et al.^[^ ^23^ ^]^ introduced a small and positive charged molecule of tris(2‐aminoethyl) amine (TAEA) to pillar the interlayer for constructing the pillared (MXene/TAEA)*_n_* multilayer composites, which shows outstanding electrical conductivity of 7.3×10^4^ S m^–1^ (Figure 1B). Moreover, several LbL assembly techniques with different diffusion‐driven kinetics, such as spinning, spraying, dipping and so forth.^[^ ^35^ ^]^ Weng et al.^[^ ^24^ ^]^ demonstrated spin spray layer‐by‐layer (SSLbL) fabrication method to rapidly prepare MXene‐CNT nanocomposite films by an apparatus. Moreover, Lipton et al.^[^ ^25^ ^]^ demonstrated the PVA‐Montmorillonite (MTM)‐MXene nanocomposite films via dip‐LbL with high strength and adjustable conductivity. VAF and LbL methods are both easy to use and can adjust the thickness by varying the concentration. However, there are still some limitations. For example, the size of the nanocomposites film is limited by the size of the filter. Hence, fabricating the larger size of nanocomposite films needs some new techniques. The scalable method of blade coating is widely used for fabricating nanocomposite films and coatings in laboratory and industrial purposes. The thickness of nanocomposite films and coatings can be adjusted through the scraping blade interval and the concentration of the slurry. For example, Zhang et al.^[^ ^26^ ^]^ fabricated the MXene films with high‐oriented MXene flakes via a blade coating technique, enhancing the degree of alignment of the MXene nanosheets by controlling the coating speed and the applied shear (Figure 1C). Compared with the laboratory‐scale VAF MXene film, the size of the obtained MXene films can reach up to meter level. Moreover, Lipton et al.^[^ ^27^ ^]^ fabricated the free‐standing MXene films via drop‐casting, and the hydrophobic plastic substrates were used to rapidly fabricate large area film with 125 cm^2^.

## 3 PROPERTIES AND APPLICATIONS OF MXene BASED NANOCOMPOSITE FILMS

### 3.1 Mechanical properties

**FIGURE 2:**
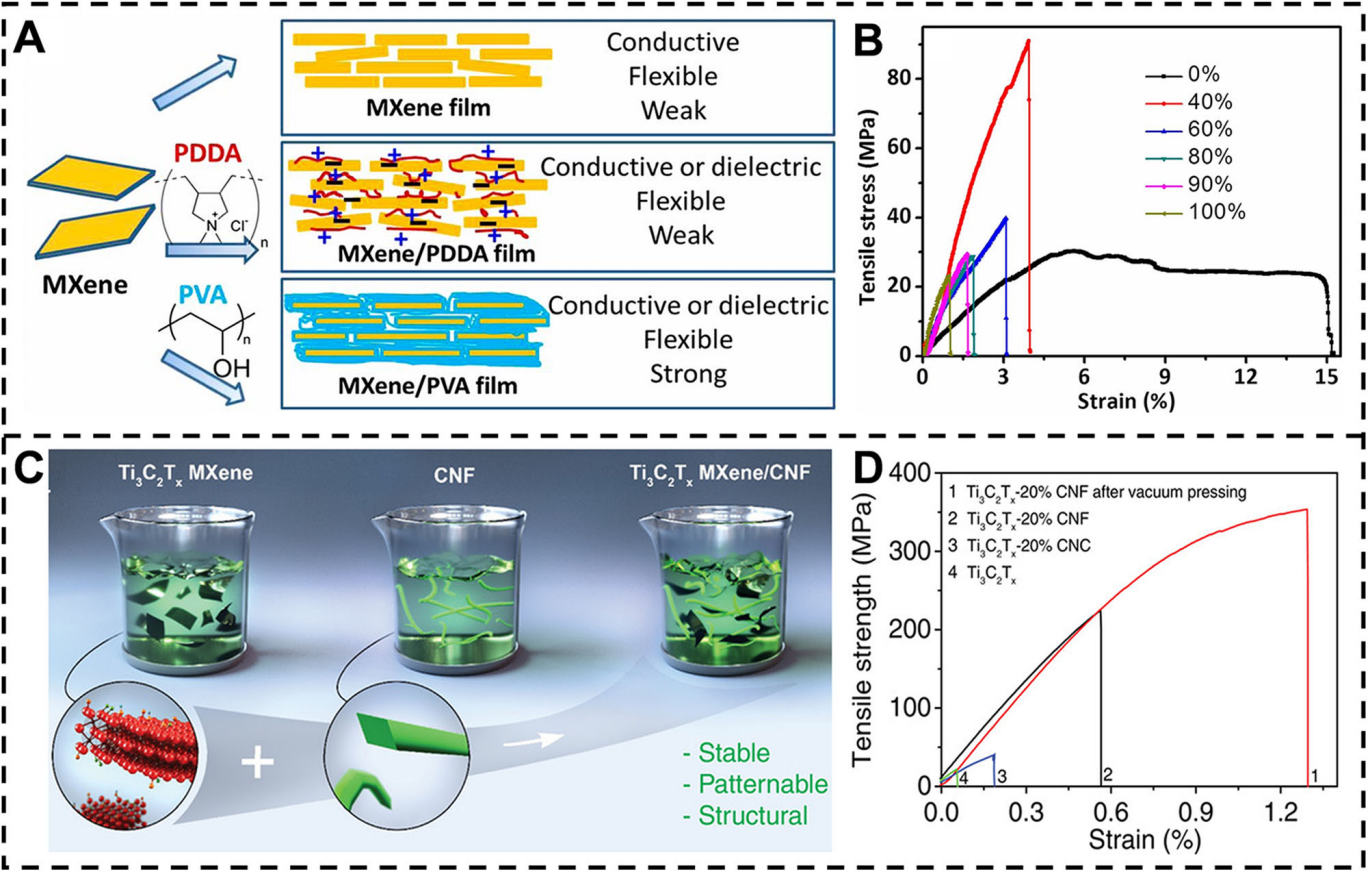
Mechanical properties of MXene‐based nanocomposite films. (A) Schematic diagram of pure MXene film and MXene‐based nanocomposite films. (B) Stress–strain curves of MXene‐PVA nanocomposite films. Reproduced with permission.^[^ ^12^ ^]^ Copyright 2014, National Academy of Sciences. (C) Schematic diagram of MXene‐CNF hybrids dispersion. (D) Stress–strain curves of MXene‐CNF nanocomposite films before and after vacuum pressing. Reproduced with permission.^[^ ^18^ ^]^ Copyright 2019, Wiley‐VCH

Mechanical properties are most important parameters for MXene‐based nanocomposite films in many application fields. For instance, Ling et al.^[^ ^12^ ^]^ prepared MXene composite films via a simple VAF process for the first time (Figure 2A). Two kinds of polymers such as PDDA and PVA are introduced into the MXene to fabricate the conductive and free‐standing MXene nanocomposite films. The PDDA is a cationic polymer while the MXene flakes are negatively charged, which can easily interact with MXene flakes. In addition, the PVA shows the water solubility and many hydroxyl groups, which can adjust the spacing between MXene flakes. The tensile stress–strain curves of MXene‐PVA nanocomposite films show that the tensile strength increases with decreasing content of MXene (Figure 2B). The tensile strength of MXene‐PVA nanocomposite films reaches up to 91 MPa with 60 wt% PVA, which is around four times higher than pure MXene film (∼22 MPa). Moreover, compared to PVA, the CNF can easily disperse and stabilize the electronic materials such as MXene and be used as the binders for fabricating nanocomposite films. Tian et al.^[^ ^18^ ^]^ reported the MXene‐CNF nanocomposite films with high mechanical and electrochemical properties via VAF and vacuum pressing process (Figure 2C). The nanocomposite films after vacuum pressing synchronously integrate a high strength of 341 MPa and Young's modulus of 42.8 GPa, which is 1.6 times higher than the initial MXene‐CNF nanocomposite films, because the forming of stronger interconnection and dense structure of MXene‐CNF nanocomposite film can reduce the voids using vacuum pressing (Figure 2D). Some other fillers, such as ANF,^[^ ^29^ ^]^ PDA,^[^ ^16^ ^]^ and BC,^[^ ^31^ ^]^ are also introduced into the MXene to enhance the mechanical properties of nanocomposites. So far, most of MXene‐based nanocomposite films show poor mechanical properties even though adding a mass of fillers. Hence, the MXene flake size, orientation, and compaction are needed to be improved to further reinforce the mechanical and electrical properties of MXene‐based nanocomposites. For example, Zhang et al.^[^ ^26^ ^]^ demonstrated the outstanding mechanical and electrical conductivity properties of MXene films via a blade coating technique. The general lateral dimension and thickness of MXene flakes is around 10 μm and 1.7 nm, and the aspect ratio reaches up to 5880, which leads to lyotropic liquid crystals of high concentration MXene nanosheets dispersion liquid. Then, the obtained MXene films with high alignment of flakes show high strength of 568 MPa, Young's modulus of 20.6 GPa, and electrical conductivity of 12300 S cm^–1^, respectively. Table 1 exhibits the mechanical properties of different MXene‐based nanocomposites reported so far.^[^ ^12^, ^15^, ^16^, ^17^, ^18^, ^19^, ^20^, ^21^, ^22^, ^24^, ^25^, ^26^, ^28^, ^29^, ^30^, ^32^, ^36^, ^37^, ^38^, ^39^ ^]^

**TABLE 1:** Mechanical properties of MXene‐based nanocomposite films

**MXene‐based nanocomposites**	**Filler**	**Content (wt%)**	**Strength (MPa)**	**Toughness (MJ m^–3^)**	**Modulus (GPa)**	**Conductivity (S m^−1^)**	**Method**	**Ref**.
MXene‐PVA	PVA	60	91	1.83	3.7	0.04	VAF	[12]
MXene‐PEDOT:PSS	PEDOT:PSS	25	30	0.225	1.9	2040	VAF	[30]
MXene‐CNT	CNT	/	25	0.05	6.25	13000	SSLbL	[24]
MXene‐CNF	CNF	50	135.4	14.8	3.8	9.691	VAF	[28]
MXene‐GO	GO	50	209	1.09	16.9	46200	VAF	[32]
MXene‐MTM‐PVA	MTM/PVA	/	225	2.25	10.5	125	LbL	[25]
MXene‐TONCF	TOCNFs	60	212	5.5	5.9	1212	VAF	[21]
MXene‐ANF	ANF	90	197.1	12.4	2.01	29.5	VAF	[29]
MXene‐CNT‐CNF	CNT/CNF	25	97.9	2.1	2.6	2506	VAF	[22]
MXene‐CNF	CNF	20	341	2.73	42.8	22500	VAF/Vacuum pressing	[18]
MXene	/	/	112	1.48	4.48	1040000	VAF/HCl treated	[15]
MXene‐ANF	ANF	20	160	4.3	4.5	/	VAF	[19]
MXene	/	/	568	10.2	20.6	1150000	Blade coating	[26]
SBM	Ca^2+^/SA	/	436	8.39	14	298800	VAF	[17]
MXene‐PDA	PDA	5	237	4.38	6.8	432296	VAF	[16]
MXene‐CNF	CNF	50	112.5	2.7	/	82	Alternating VAF	[36]
ANF‐MXene‐AgNW	ANF	80	235.9	30	/	92200	VAF/Hot pressing	[37]
PDMM‐BCNF	PDA/BCNF	40	406	15.3	7.5	284800	VAF	[38]
SBM	CMC/Boron	10	583	15.9	27.8	611500	VAF	[20]
MXene‐BC	BC	74.3	297.5	14.9	5.1	/	In situ biosynthesis	[39]

Cheng's group from Beihang University proposed the sequential bridging strategy to assemble MXene flakes into high performance nanocomposite films. For example, Wan et al.^[^ ^17^ ^]^ demonstrated the strong and high conductivity of MXene‐based nanocomposite films through a sequential bridging of hydrogen bonding and ionic bonding. First, the MXene flakes were bridged with SA through hydrogen bonding, and then the calcium ion (Ca^2+^) was introduced into the MXene‐SA hybrid to fabricate the obtained sequentially bridged (SBM) MXene nanocomposite films. The resultant SBM nanocomposite films exhibit the high strength of 436 Mpa, Young's modulus of 14 Gpa, and the toughness of 8.39 MJ m^−3^, respectively, which are 6.9, 13.5, and 2.5 folds higher than those for pure MXene film.

**FIGURE 3:**
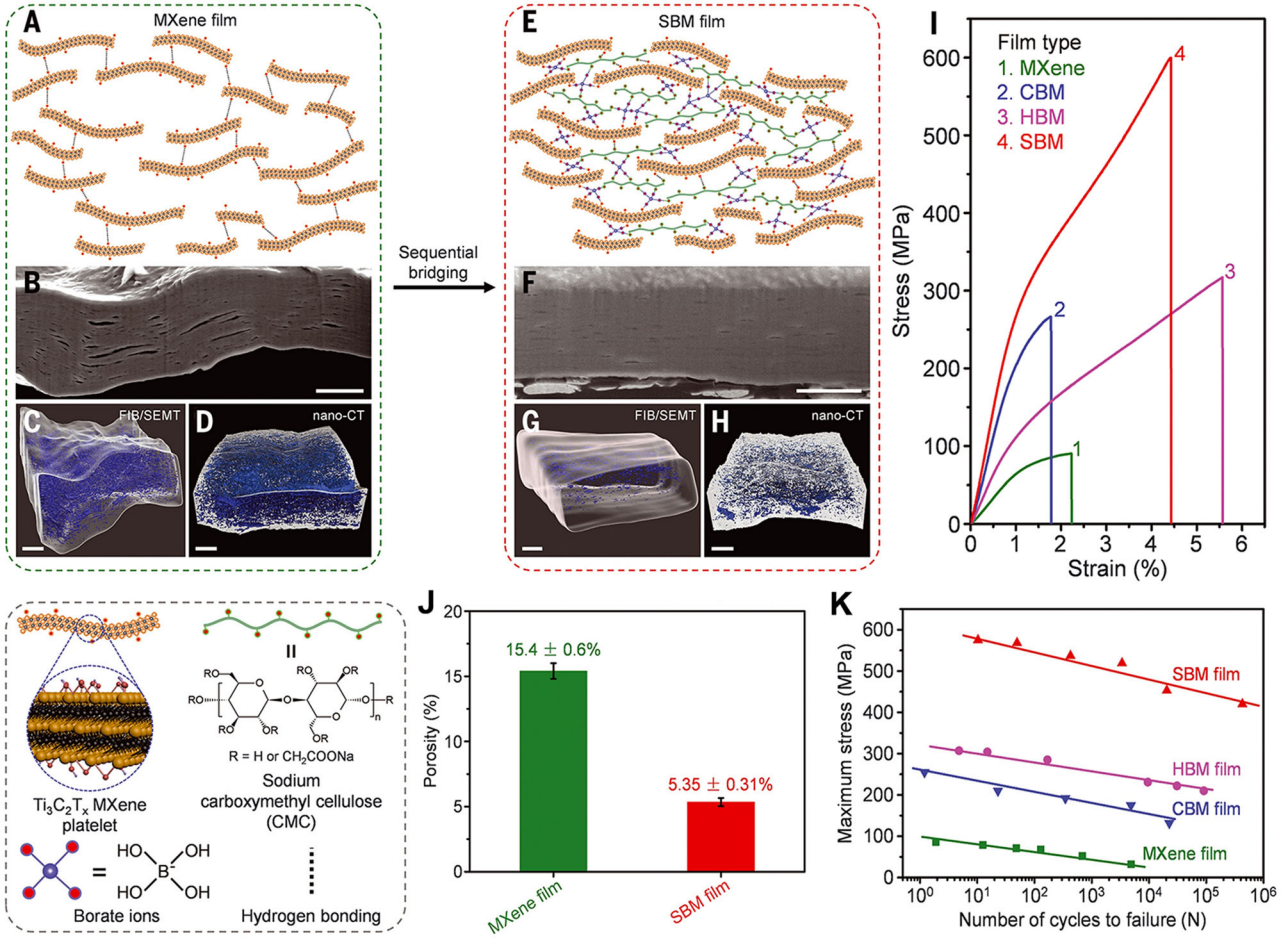
Sequential bridging high strength MXene‐based nanocomposite films. (A, E) Model structure representation of pure MXene film and SBM nanocomposite film. (B, F) Cross‐sectional SEM images cut by FIB for pure MXene film and SBM nanocomposite film. (C, G) Three‐dimensional remodeled void microstructure root in FIB/SEMT for pure MXene film and SBM nanocomposite film. (D, H) Three‐dimensional remodeled void microstructure root in nano‐CT for pure MXene film and SBM nanocomposite film. (I) Representative stress–strain curves of pure MXene film, covalent bonding MXene (CBM), hydrogen bonding MXene (HBM), and SBM nanocomposites. (J) Porosities of pure MXene film and SBM nanocomposite film root in density measurements. (K) Variations of maximum applied stress of MXene, CBM, HBM, and SBM films after manifold cycles fatigue test. Reproduced with permission.^[^ ^20^ ^]^ Copyright 2021, AAAS

Recently, Cheng's group demonstrated that the existing voids of MXene nanocomposite films are an obstacle to obtain high performance MXene nanocomposite films. There are three pivotal factors for assembling high performance macroscopic MXene nanocomposites films: interface effect, alignment, and compactness. Hence, Cheng's group^[^ ^20^ ^]^ developed an efficient densification strategy to eliminate the voids through hydrogen and covalent bonding (Figure 3A,E). The CMC is first introduced into the MXene flakes through hydrogen bonding to form a freestanding hydrogen‐bonded MXene (HBM) film, and then the small borate ions are infiltrated into HBM film to covalently crosslink with ‐OH of CMC molecule and MXene flakes. The voids microstructure of original MXene and SBM films have been remodeled by focused ion beam (FIB) and SEM tomography and nanoscale x‐ray computed tomography (Figure 3B–H). The SBM films have denser flake stacking and fewer voids than the MXene films. The porosity of the SBM film shows only 5.35 ± 0.3%, which is lower than the pure MXene film of 15.4 ± 0.6% (Figure 3J). Due to the synergistic densification induced, the SBM films exhibit the highest strength of 583 MPa, Young's modulus of 27.8 GPa, and toughness of 15.9 MJ m^–3^, respectively, which are 5.7, 11.2, and 3.6 folds higher than those for pure MXene film (Figure 3I). Moreover, SBM films exhibit excellent fatigue resistance and remain 69.5% maximum stress after 420,000 cycles stretching, however, the pure MXene film only remain 36.7% maximum stress after 4854 cycles stretching (Figure 3K).

### 3.2 EMI shielding

**FIGURE 4:**
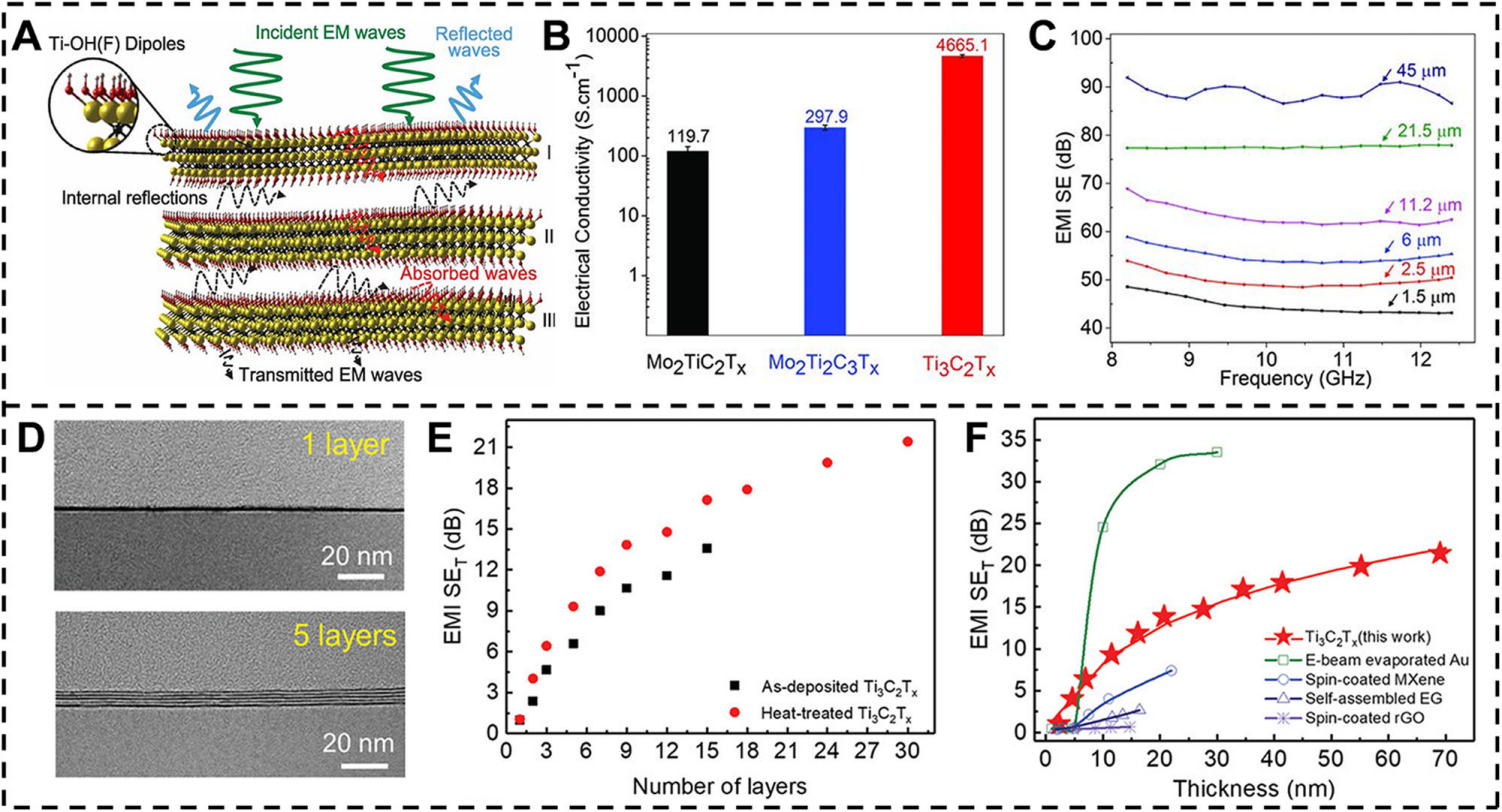
EMI shielding performance of pure MXene films. (A) Schematic diagram of MXene EMI shielding mechanism. (B) Electrical conductivity of three kinds of MXenes of Mo_2_TiC_2_T*_x_*, Mo_2_Ti_2_C_3_T*_x_*_,_ and Ti_3_C_2_T*_x_*. C EMI SE of Ti_3_C_2_T*_x_* MXene film with different thickness. Reproduced with permission.^[^ ^9^ ^]^ Copyright 2019, AAAS. (D) TEM images of monolayer and five layers of MXene film. (E) EMI SE of different layers of MXene film before and after 400°C heat treatment. (F) Comparison of EMI SE at different thickness for several EMI shielding materials. Reproduced with permission.^[^ ^42^ ^]^ Copyright 2020, Wiley‐VCH

With the miniaturized and multi‐frequency development of the wearable electronic devices, EMI pollution has aroused more consideration. Traditionally, metal‐based and polymer‐based composites have been generally utilized in EMI shielding field. However, easy corrosion, high density, and inflexibility of metal‐based materials and the millimeter‐scale thickness of polymer‐based composites materials have restricted their applications in many areas.^[^ ^40^ ^]^ Moreover, the carbon materials, especially graphene, have been widely used in EMI shielding application in recent year, however, most of the high‐performance graphene films are prepared by high temperature treatment, which is hard to realize in actual massive production and limits their effective application.^[^ ^41^ ^]^ Because of the outstanding conductivity and abundant surface groups, MXenes have been considered the prospective candidate for preparing EMI shielding materials. Shahzad et al.^[^ ^9^ ^]^ first demonstrated three kinds of MXenes and their composite films for EMI shielding via simple VAF technique. The Ti_3_C_2_T*_x_* MXene shows the highest electrical conductivity of 4600 S cm^–1^, compared to 300 and 120 S cm^–1^ for Mo_2_TiC_2_T*_x_* and Mo_2_Ti_2_C_3_T*_x_*, respectively (Figure 4B).

In addition, the electrical conductivity is a considerable essential in EMI shielding. Therefore, the Ti_3_C_2_T*_x_* MXene film shows outstanding EMI shielding effectiveness (SE) of 48–92 dB with thickness of 1.5–45 μm in 8.2–12.4 GHz (Figure 4C). The EMI shielding mechanism of MXene film is illustrated in Figure 4A. When the electromagnetic waves (EMWs) interact with the MXene flakes, some of incident EMWs are promptly reflected because of the ultrahigh conductivity of MXene flakes. The other EMWs pass through the MXene layer and are attenuated of energy by interacting with high electron concentration of MXene. Then, the residual EMWs pass through next MXene layer and repeat the attenuation. Subsequently, the EMWs are then reflected between the adjacent MXene layers until they are fully absorbed. Moreover, the nacre‐structure of the MXene film will reinforce the multiple interfacial reflection. In addition, for eliminating the influence of thickness on EMI shielding performance, the absolute EMI SE effectiveness (SSE_t_) of materials is defined as SE divided by the thickness and density. The SSE_t_ of MXene film is 25863 dB cm^2^ g^–1^, which is larger than the Cu foil of 7812 dB cm^2^ g^–1^. Except for the micrometer‐thick MXene film, the EMI shielding performance of nanometer‐thick MXene film is investigated (Figure 4D). For example, Yun et al.^[^ ^42^ ^]^ demonstrated the ultrathin MXene film via a rapid interfacial self‐assembly. The 55‐nm‐thick MXene film containing 24 layers shows an EMI SE of 20 dB, a 99% shielding efficiency (Figure 4E,F). For 3‐layer MXene film, the SSE_t_ shows a maximum value of 3,890,000 dB cm^2^ g^−1^, which is quite a high value in the previous report.

**FIGURE 5:**
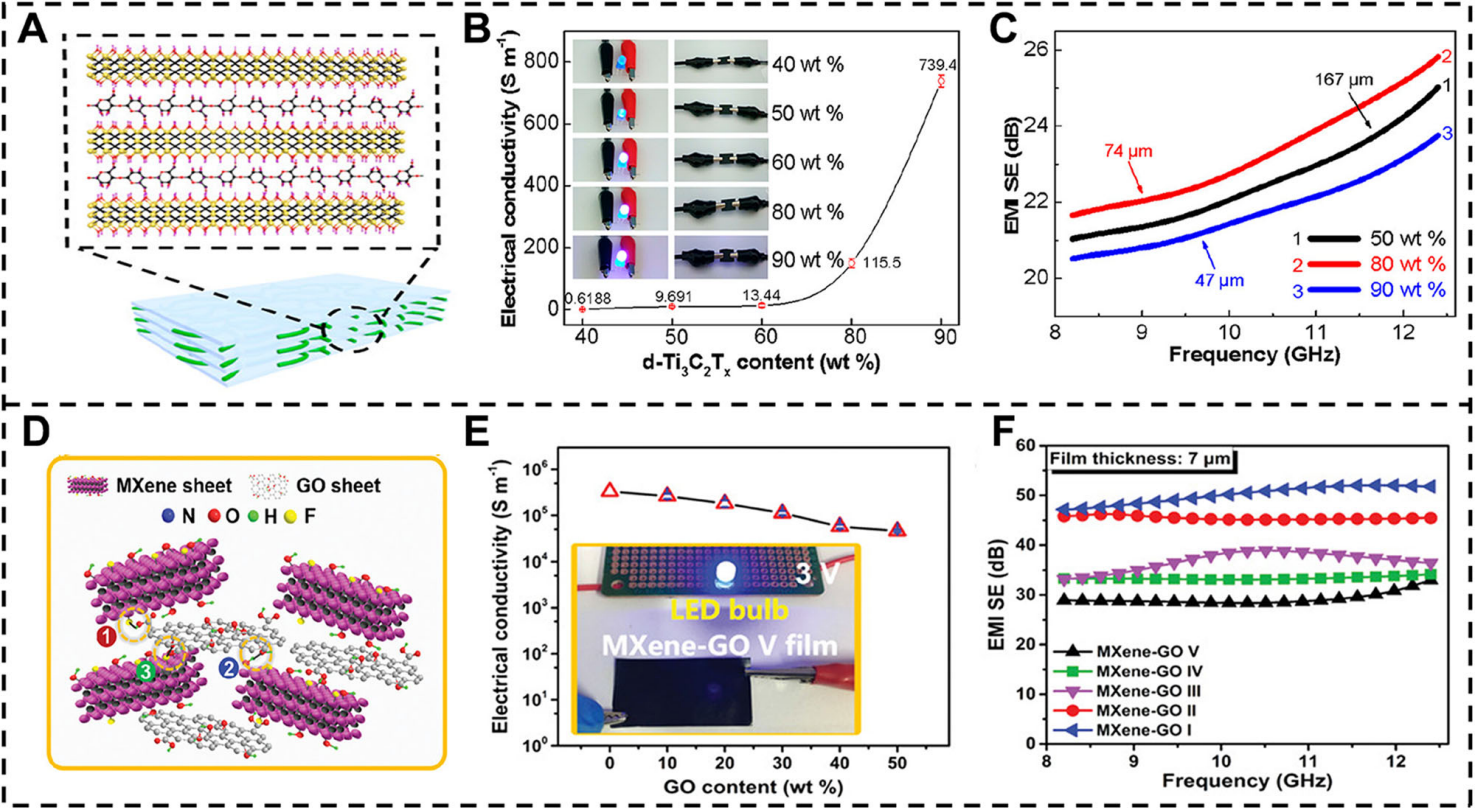
EMI shielding performance of MXene nanocomposite films. (A) Schematic diagram of the MXene‐CNF nanocomposite films. (B) Electrical conductivity of MXene‐CNF nanocomposite films. (C) EMI SE of MXene‐CNF nanocomposite films with different MXene content. Reproduced with permission.^[^ ^28^ ^]^ Copyright 2018, American Chemical Society. (D) Schematic diagram of the MXene‐GO nanocomposite films. (E) Electrical conductivity of MXene‐GO nanocomposite films. (F) EMI SE of MXene‐GO nanocomposite films. Reproduced with permission.^[^ ^32^ ^]^ Copyright 2019, Wiley‐VCH

The researches of EMI shielding performance of MXene‐based nanocomposite films have attracted enormous attention. For example, nacre‐like MXene‐CNF nanocomposite films have been fabricated with VAF technique, and the EMI SE and mechanical properties are investigated (Figure 5A).^[^ ^28^ ^]^ The 47‐μm‐thick nanocomposite film with adding 80 wt% MXene shows an EMI SE of 25.8 dB, SSE_t_ of 2154 dB cm^2^ g^−1^, and tensile strength of 60.2 MPa, respectively (Figure 5B,C). However, these fillers are electrical insulating and the conductivity of composites is inevitably deteriorated, resulting in poor EMI shielding performance. Hence, conductive fillers are used in fabricating MXene‐based nanocomposite films with outstanding EMI shielding performance. Liu et al.^[^ ^30^ ^]^ prepared the flexible and nacre‐like nanocomposite films using MXene and PEDOT:PSS via a simple VAF technique. When the MXene content increases, the EMI SE of MXene‐PEDOT:PSS is increased and reaches up to 42.1 dB with adding 82.5 wt% MXene, and a SSE_t_ of 19497.8 dB cm^2^ g^–1^ is gained. Moreover, Liu et al.^[^ ^32^ ^]^ prepared the MXene‐based nanocomposite films with adding GO via a VAF technique and hydroiodic acid (HI) treatment reducing method (Figure 5D). The MXene‐GO nanocomposite film with adding 50 wt% GO shows an electrical conductivity of 461 S m^−1^ and EMI SE of 29 dB, respectively (Figure 5E,F). After HI reducing treatment, the MXene‐rGO nanocomposite film shows a higher conductivity of 500 S m^−1^ and EMI SE of 40 dB, respectively. Furthermore, Table 2 summarizes the EMI shielding performance of different MXene‐based nanocomposite films reported so far.^[^ ^9^, ^10^, ^15^, ^16^, ^17^, ^20^, ^21^, ^22^, ^24^, ^25^, ^26^, ^28^, ^29^, ^30^, ^33^, ^36^, ^37^, ^39^, ^43^ ^]^

**TABLE 2:** Comparison of EMI SE. EMI SEt versus different MXene‐based nanocomposite films

**MXene‐based nanocomposites films**	**SE (dB)**	**Thickness (μm)**	**SSE/t (dB cm^2^ g^−1^)**	**Ref**.
MXene	68	11	25863	[9]
MXene‐SA	57	8	30830	[9]
MXene‐CNF	25	16.7	1326	[28]
MXene‐PEDOT:PSS	42.1	11	19497	[30]
MXene‐CNT	2.7	0.17	58187	[24]
MXene‐TONCF	32.7	47	4761	[21]
MXene‐ANF	30	17	13176	[29]
HCl‐processed MXene	60.4	3	51624	[15]
MXene‐CA	54.3	26	17586	[43]
MXene‐MTM‐PVA	20	3	24550	[25]
MXene‐PVA	44.4	27	9343	[10]
MXene‐CNT‐CNF	38.4	38	8020	[22]
CNF‐MXene	39.6	35	7029	[36]
ANF‐MXene/Agnws	57.3	50	9317	[37]
Blade coated MXene	53.5	2.4	50872	[26]
MXene‐PDA	58.4	6.95	83999	[16]
MXene‐SA‐Ca^2+^	46.2	2.8	58929	[17]
MXene‐MMT	67	25	10156	[33]
MXene‐BCs	26	5.2	22857	[39]
SBM	56.4	3	62458	[20]

### 3.3 Thermal conductivity

**FIGURE 6:**
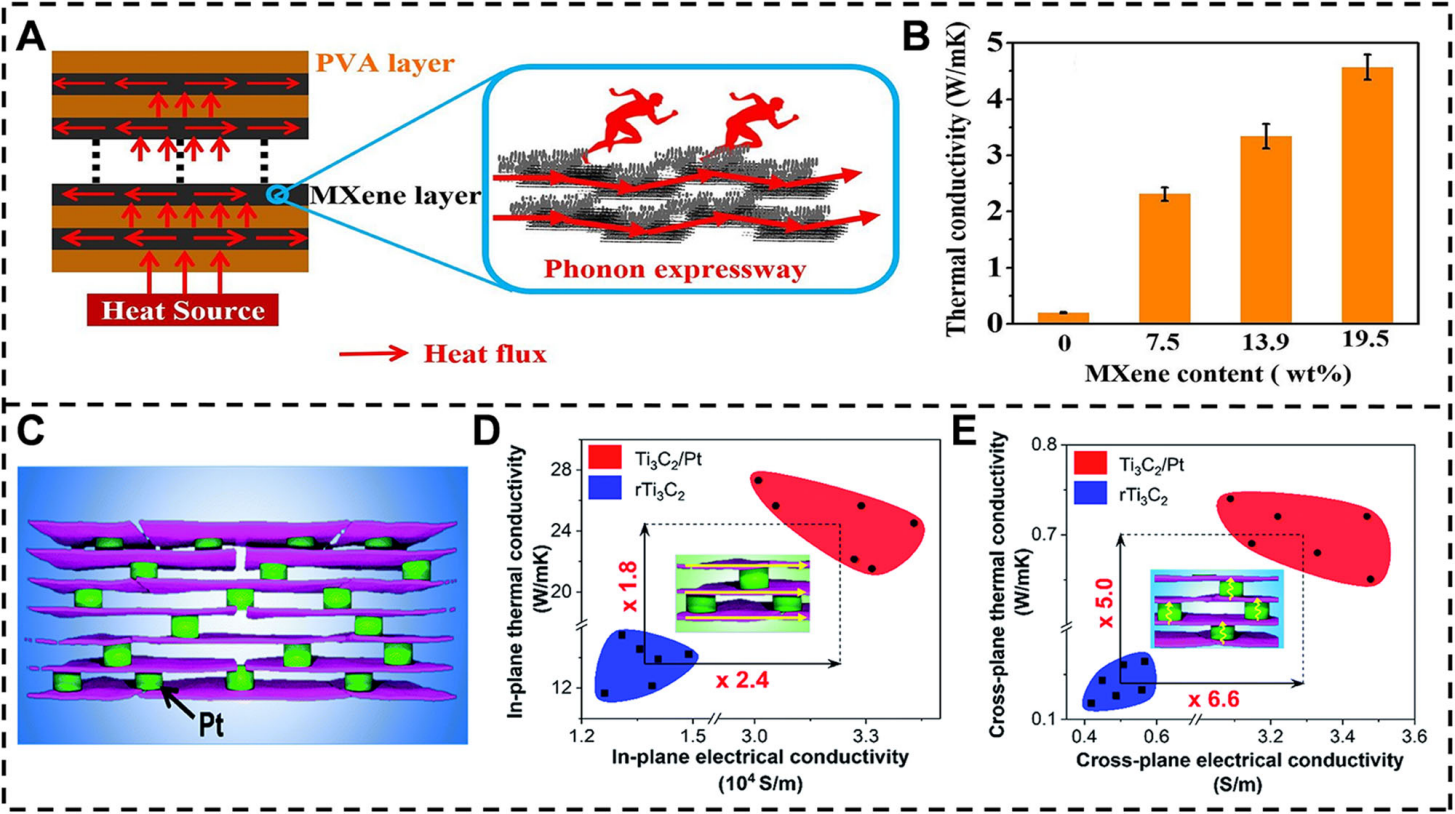
Thermal conductivity of MXene‐based nanocomposite films. (A) Schematic diagram of the thermal conductivity mechanism of MXene‐PVA multilayered nanocomposite films. (B) Thermal conductivity of pure PVA and MXene‐PVA nanocomposite films. Reproduced with permission.^[^ ^10^ ^]^ Copyright 2019, Elsevier. (C) Schematic diagram of the MXene‐Pt nanocomposite films. (D, E) In‐plane and cross‐plane thermal conductivity and electrical conductivity of pure MXene and MXene‐Pt films. Reproduced with permission.^[^ ^45^ ^]^ Copyright 2021, Royal Society of Chemistry

Because of its 2D structure like graphene, the MXene also shows excellent thermal conductivity. The researches on thermal conductivity of MXene mostly focus on theoretical simulation. For instance, Gholivand et al.^[^ ^8^ ^]^ calculated that the thermal conductivity of Ti_3_C_2_T*_z_* MXene reaches up to 108 W m^–1^ K^–1^ with fluorine‐terminated using density functional theory (DFT). In addition, Zha et al.^[^ ^44^ ^]^ calculated the thermal conductivity of Sc_2_CT_2_ MXene with 472 W m^−1^ K^−1^, which exhibits tremendous potential for fabricating high thermal conductivity MXene‐based nanocomposite films. Recently, the thermal conductivity of MXene‐based nanocomposite films have been studied. For example, Jin et al.^[^ ^10^ ^]^ demonstrated the MXene‐PVA multilayered nanocomposite films via alternating drop casting approach. The in‐plane thermal conductivity of MXene‐PVA nanocomposite films is increased with increasing MXene content. The 27‐μm‐thick MXene‐PVA nanocomposite film with adding 19.5 wt% MXene shows a high thermal conductivity of 4.57 W m^−1^ K^−1^, which is 23 times higher than the PVA film (Figure 6B). As demonstrated in Figure 6A, the MXene layer provides several continuous thermal conductive channels in the multilayer films, which makes the phonon quickly transit. Furthermore, the adjacent MXene flakes are overlapped in the MXene layer, resulting in low interface thermal resistance and effective phonon conduction pathways. Moreover, Li et al.^[^ ^33^ ^]^ prepared the MXene‐MMT nanocomposite films via a simple VAF technique, and the aligned nacre‐like structure endows the nanocomposite films with an outstanding anisotropic thermal conductivity performance. The in‐plane and cross‐plane thermal conductivity of MXene‐MMT nanocomposite films is 28.8 and 0.27 W m^−1^ K^−1^, respectively, demonstrating the excellent anisotropic thermal conductivity. As aforementioned, the existing voids of MXene‐based nanocomposites are deteriorating various performance of nanocomposites. Hence, Nguyen et al.^[^ ^45^ ^]^ demonstrated the MXene‐platinum (Pt) nanocomposite films via the atomic layer deposition (ALD). The vapor phase infiltration phenomenon of Pt can heal the defects and be linked with covalent bonding from MXene flakes, respectively. The MXene‐Pt nanocomposite films show a high in‐plane and a cross‐plane thermal conductivity of 24.45 and 0.14 W m^−1^ K^−1^, which is 1.8 and 5 folds higher than pure MXene film of 13.6 and 0.7 W m^−1^ K^−1^, respectively.

In addition, as shown in Table 3, several MXene‐based thermal conductive nanocomposite films are summarized.^[^ ^10^, ^33^, ^45^, ^46^, ^47^, ^48^, ^49^, ^50^ ^]^ Up to now, the thermal conductivity of MXene‐based nanocomposite films is still relatively low. Hence, how to construct the high thermal conductivity MXene‐based nanocomposite films is still a challenge. The alignment and compactness of the structure may play a vital role in fabricating high thermal conductive MXene‐based nanocomposite films, because the existing voids increase the interface resistance and lead to low phonon transit efficiency.

**TABLE 3:** Thermal conductivity of different MXene‐based nanocomposite films

**MXene‐based nanocomposite films**	**Content (wt%)**	**TC (W mK^−1^)**	**Testing method**	**Ref**.
MXene‐PVA	19.5	4.57	Laser flash	[10]
MXene‐MMT	90	28.8	Laser flash	[33]
CNF‐MXene	50	11.57	Laser flash	[46]
CNF‐MXene‐Agnws	35	15.53	Laser flash	[47]
GO‐MXene	40	11.24	Laser flash	[48]
Graphene‐MXene	40	26.49	Laser flash	[48]
CNF‐MXene‐Ag	30	22.43	Laser flash	[49]
CNF‐MXene	60	14.93	Laser flash	[50]
MXene‐Pt	/	24.45	Laser flash	[45]

### 3.4 Supercapacitor

**FIGURE 7:**
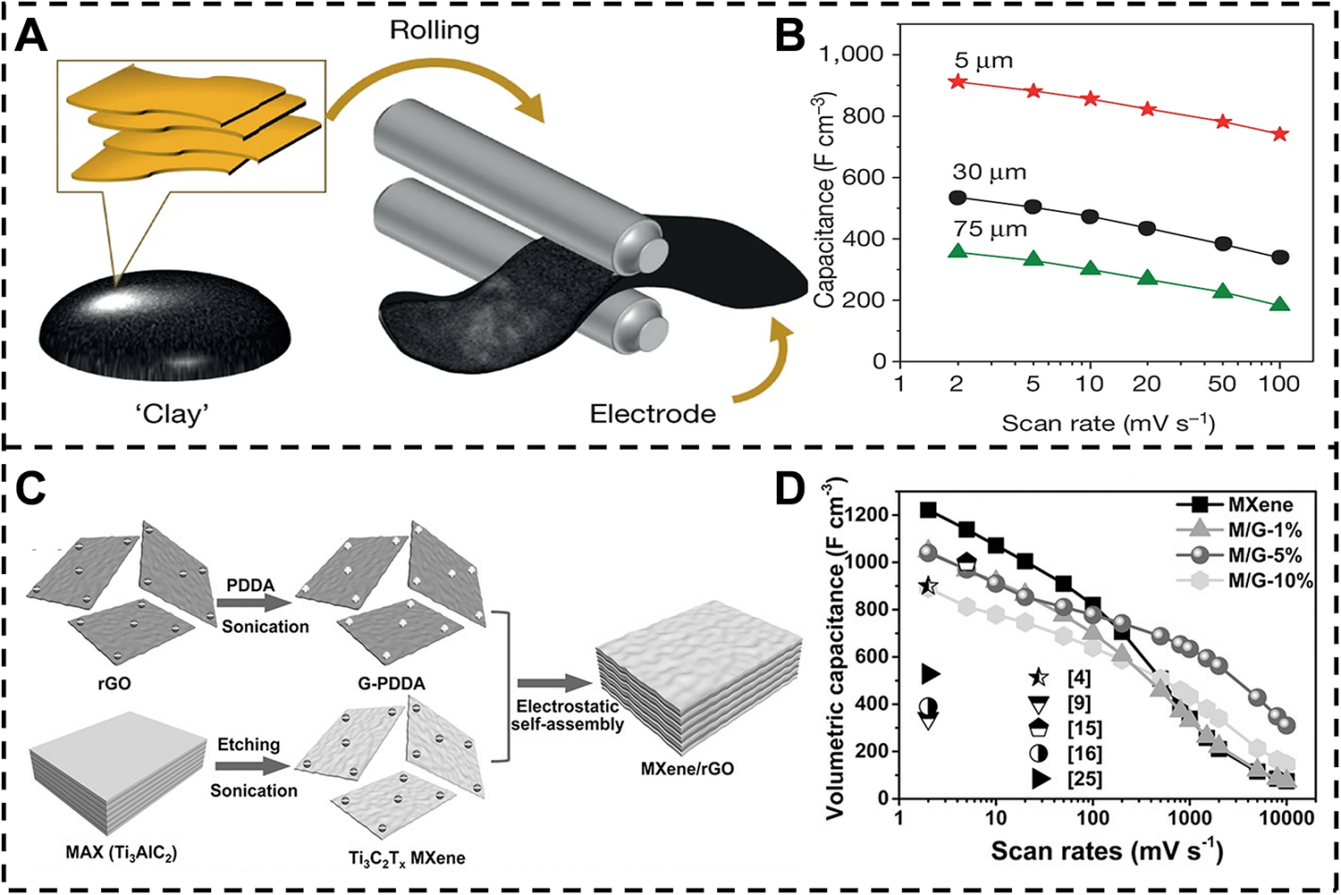
Supercapacitor application of MXene‐based nanocomposite films. (A) Schematic illustration of the MXene clay electrode preparation. (B) Capacitances of different thickness MXene clay electrodes. Reproduced with permission.^[^ ^56^ ^]^ Copyright 2014, Springer Nature. (C) Schematic illustration of the MXene‐rGO nanocomposite films. (D) Volumetric capacitances of MXene and MXene‐rGO nanocomposite electrodes with different GO content. Reproduced with permission.^[^ ^11^ ^]^ Copyright 2017, Wiley‐VCH

With the widespread use of portable and wearable devices, flexible energy storage devices have experienced rapid development.^[^ ^51^, ^52^, ^53^ ^]^ Generally, the supercapacitors show outstanding power density and high cycle efficiency, which is an ideal candidate for wearable electronics.^[^ ^54^, ^55^ ^]^ Recently, because of their high conductivity and hydrophilicity, MXenes have been demonstrated as potential candidates for supercapacitors. For instance, Gogotsi's group^[^ ^7^ ^]^ firstly reported the volumetric capacitance of MXene films. In some electrolytes such as KOH and NaOAc, the volumetric capacitance is 340 F cm^−3^ at a scan rate of 2 mV s^−1^ for few‐layer MXene electrode, which is two folds higher than the multilayer MXene electrode. Moreover, Ghidiu et al.^[^ ^56^ ^]^ prepared the additive‐free films of ‘‘clay’’ with high volumetric capacitances and gravimetric capacitances of 900 F cm^−3^ and 245 F g^−1^ at a scan rate of 2 mV s^−1^ via a rolling technique (Figure 7A,B). Moreover, the volumetric capacitances are decreasing with increased thickness of the film. Even after 10,000 cycles, the capacitance loss is almost zero. Then, the vertical arrangement of MXene flakes of MXene films is prepared using mechanical shearing liquid crystalline phase of MXene.^[^ ^57^ ^]^ The films show the excellent retention capacitance over 200 F g^−1^ even though the thickness of the films with 200 μm at a high scan rate of 2,000 mV s^–1^.

Typically, owing to restacking of the nanosheets, the electrodes composed of laminar nanomaterials undergo limited electrolyte‐accessible surface area, which decreases the energy density and electrical conductivity. Hence, several conductive materials such as polypyrrole (PPy),^[^ ^13^ ^]^ carbon nanotube (CNT),^[^ ^58^ ^]^ and graphene^[^ ^11^ ^]^ have been introduced into the MXene to effectively prevent the restacking. For instance, Yan et al.^[^ ^11^ ^]^ demonstrated the MXene‐graphene supercapacitor electrode films through an electrostatic self‐assembly technique using the positive rGO‐PDDA and negative MXene (Figure 7C). The rGO nanosheets are introduced into the MXene, contributing to the increased interlayer spacing of MXene. Then, MXene‐rGO film electrode with adding 5 wt% rGO shows a volumetric capacitance of 1040 F cm^–3^ at a scan rate of 2 mV s^−1^, and the remain 61% capacitance retention at 1 V s^−1^ after 20,000 cycles (Figure 7D). Moreover, the conducting polymer of PPy is bonded with MXene to fabricate the PPy‐MXene film with a high volumetric capacitance of 406 F cm^–3^ and outstanding stability.^[^ ^13^ ^]^ Furthermore, Zhao et al.^[^ ^58^ ^]^ prepared the sandwich‐like MXene‐CNT nanocomposite paper with a volumetric capacitance of 390 F cm^–3^ at a scan rate of 2 mV s^−1^ via alternating VAF technique.

## 4 SUMMARY AND OUTLOOK

Since first report of MXene in 2011, the past decade has witnessed a rapid progress in the researches of MXene‐based nanocomposite films due to the excellent physicochemical properties of MXene. This review has concentrated on the fabrication approach, mechanical and electrical properties of MXene‐based nanocomposite films. Moreover, we summarize the functional applications of MXene‐based nanocomposite films such as EMI shielding, thermal conductivity, and supercapacitor. There are various methods for preparing MXene‐based nanocomposite films such as VAF, LbL, blade coating, and drop‐casting. Based on these methods, several MXene‐based nanocomposite films have been successfully fabricated. The MXene‐based nanocomposite films show the high mechanical, electrical, and thermal conductivity properties and are widely applied in EMI shielding, heat dissipation, and supercapacitor. Despite having achieved great progress, several questions and challenges of MXene‐based nanocomposite films exist and need to be addressed until their practical applications.

- The existing voids of MXene‐based nanocomposite films are inevitably deteriorating the mechanical properties. Hence, some new strategies have been investigated to reduce the voids in MXene‐based nanocomposite films. For example, the blade coating fabrication process improve the alignment degree of MXene flakes, leading to a high tensile strength. The strong covalent and ionic interfacial interactions decrease the existing voids, which can reach high strength and toughness. Hence, several factors such as the compactness, interlayer interaction, and alignment of MXene flakes, play a vital role in achieving high mechanical properties of MXene‐based nanocomposite films. Recently, the sequential bridging strategy has been proposed to further reduce the existing voids, which is useful for fabricating high performance MXene‐based nanocomposites. Furthermore, other approaches, such as filling small size flakes or molecules and external forces inducing are needed to be explored. Meanwhile, the preparing methods of MXene are needed to be investigated, and can adjust the layer structure and surface groups of MXene, which can improve the mechanical properties of MXene and its nanocomposite films.
- Even though the EMI shielding performance of MXene‐based nanocomposite films is relatively high, the poor oxidation‐resistant and high temperature‐resistant properties limit the practical applications of MXene‐based nanocomposite films. Moreover, the pure MXene films show a low tensile strength. Various kinds of insulating fillers such as polymer and small molecular are introduced into the MXene flakes to fabricate MXene‐based nanocomposite films, which may decrease the EMI shielding performance. Some new strategies have been proposed. For example, the alternating multilayer structure can enhance the EMI shielding performance and mechanical properties. Hence, the MXene‐based EMI shielding nanocomposite films with high mechanical properties, long term durability, and environmental stability during utilization should be focused on in future study.
- Due to the intrinsic thermal conductivity of MXene, MXene‐based nanocomposite films have a lot of promising potential to be used in thermal management materials. However, the practical thermal conductivity of MXene‐based nanocomposite films fall far below the theoretical thermal conductivity of MXene. Hence, how to prepare the high thermal conductivity of MXene‐based nanocomposite films is still a challenge to solve. Some vital issues are needed to be as follows. The alignment degree of MXene flakes is crucial to form effective thermal conductive pathways. Furthermore, surface terminating functional groups of MXene also can influence the thermal conductivity of MXene. Then, the high thermal conductive fillers such as BN, graphene, and metal particle are inserted into the MXene to reduce the voids in MXene‐based nanocomposite films.
- Despite the outstanding energy storage performance of MXene, there are still some questions for fabricating high performance MXene‐based supercapacitors. The main challenge is the restacking of the MXene flakes, which limits the ion transport and decreases the energy density and electrical conductivity. The several materials such as PPy, CNT, and graphene are introduced into the MXene to prevent the restacking and increase the interlayer distance. Furthermore, the architectural design of MXene‐based nanocomposites play another key role in fabricating high performance MXene‐based supercapacitors.

Due to the unique physicochemical properties and typical structure of MXene, the MXene‐based nanocomposite films have been broadly applied in many fields. However, the size of most of the MXene‐based nanocomposite films are lab‐sized (centimeter level) until now. Hence, in the case of practical applications, large‐scale (meter level) of MXene‐based nanocomposite films with excellent performance are urgently needed.

## CONFLICT OF INTEREST

The authors declare no conflict of interest.
